# Silkworm SOCS2 Differentially Promotes Multiple Steps of BmNPV Proliferation and Modulates the mRNA Expression of SOCS-STAT Network Components

**DOI:** 10.3390/insects17050503

**Published:** 2026-05-15

**Authors:** Cong Zhang, Hengchuan Xia, Qianzhu Wan, Yangyang Chen, Gaoying Xu, Jingao Wang, Liang Chen, Jin Wang, Keping Chen

**Affiliations:** School of Biological Science and Technology, Jiangsu University, Zhenjiang 212013, China; zhangcong968@163.com (C.Z.); jyjnts3@163.com (Q.W.); chenyangyang232913@163.com (Y.C.); xugaoying1104@163.com (G.X.); 17787643689@163.com (J.W.); oochen@ujs.edu.cn (L.C.); jinwang@ujs.edu.cn (J.W.); kpchen@ujs.edu.cn (K.C.)

**Keywords:** *SOCS2*, JAK-STAT pathway, BmNPV, transcriptional modulation, host-virus interaction

## Abstract

Suppressors of cytokine signaling (SOCSs) are negative feedback regulators of the JAK-STAT pathway and are often exploited by viruses to evade host antiviral immunity. Unlike other SOCS members, the role of *SOCS2* in viral infection remains poorly understood. Here, we report that overexpression of silkworm *SOCS2* isoforms, *SOCS2L* and *SOCS2S*, promotes *Bombyx mori* nucleopolyhedrovirus (BmNPV) replication at multiple stages, including viral DNA replication, late gene (*VP39*) transcription, and virion egress, while their knockdown suppresses these processes. Overexpression of SH2 domain mutants (R123Q in *SOCS2S*, R142Q in *SOCS2L*) reduced viral DNA replication to baseline and *VP39* expression below baseline, drastically decreased infectious progeny titers, but unexpectedly increased viral DNA release to wild-type levels, suggesting that the SH2 domain may differentially regulate distinct steps of viral replication. Furthermore, *SOCS2* isoforms, alone or cooperatively with BmNPV, modulate the mRNA levels of SOCS-STAT network members in an isoform-, dose-, and target-specific manner. Collectively, this study reveals for the first time the multiple proviral functions of silkworm *SOCS2* isoforms, with differential effects on distinct stages of the viral life cycle, and highlights their potential as transcriptional modulators exploited by viruses for immune evasion.

## Simple Summary

Silkworms are an important economic insect, but they are often infected by a virus called BmNPV, causing serious losses to sericulture. In this study, we discovered that a host factor, suppressor of cytokine signaling 2 (*SOCS2*), which normally negatively regulates immune responses, can be exploited by the virus to promote its own replication. Silkworms produce two forms of *SOCS2*: a long isoform (*SOCS2L*) and a short one (*SOCS2S*). Both forms promote viral multiplication at different stages of the viral life cycle. Specifically, they enhance viral DNA replication and late gene expression in an SH2 domain-dependent manner, while increasing the release of viral DNA or particles in a manner independent of the SH2 domain. Furthermore, both forms can alter the mRNA levels of other SOCS and STAT family members, either on their own or together with the virus. Notably, *SOCS2L* exhibits stronger regulatory effects than *SOCS2S* in these functions. Our findings reveal a previously unknown role of *SOCS2* as a promoter of viral infection in insects, providing new insights into how viruses hijack host factors and suggesting a potential target for controlling viral diseases in silkworms.

## 1. Introduction

The Janus kinase-signal transducer and activator of transcription (JAK-STAT) pathway is a cornerstone of antiviral immunity [1]. Its precise regulation by Suppressors of Cytokine Signaling (SOCSs), negative feedback inhibitors directly induced by STAT activation, ensures appropriate immune responses and prevents immunopathology [2,3,4]. SOCSs generally act as substrate-recognition subunits of Cullin-RING E3 ubiquitin ligase complexes: their SH2 domain binds phosphorylated signaling intermediates such as cytokine receptors, while their C-terminal SOCS box recruits the elongin B/C-Cullin5-Rbx2 complex, targeting bound proteins for ubiquitination and proteasomal degradation. Among mammalian SOCS family, *SOCS1* and *SOCS3* are frequently exploited by viruses to suppress JAK-STAT antiviral signaling [2], while roles of other SOCSs in viral infection remain poorly characterized. Notably, invertebrates generally lack *SOCS1/3*, and possess only one to three SOCS selected from *SOCS2/5/6/7* [1]. Whether viruses exploit these alternative SOCS factors to manipulate JAK-STAT signaling in invertebrates remains largely unknown.

*SOCS2* stands out for its context-dependent and often paradoxical roles. While acting as a canonical inhibitor, it also exhibits complex and seemingly contradictory effects. For instance, both overexpression and knockout of *SOCS2* cause gigantism in mice, indicating a dose-sensitive regulation [5,6]. Mechanistically, *SOCS2* can promote *SOCS1/3* degradation, indirectly amplifying cytokine signaling [7,8,9], but its physiological relevance remains unclear. This functional complexity may partly explain why the role of *SOCS2* in viral infection remains largely elusive. The limited studies available indicate that HIV and HBV upregulate *SOCS2* to suppress interferon signaling via the JAK-STAT pathway and promote their replication [10,11]. Conversely, host cells may downregulate *SOCS2* as a countermeasure [12]. In invertebrates, studies are even scarcer. Interestingly, knockdown of shrimp *SOCS2* promotes STAT activation [13] and increases resistance to the white spot syndrome virus (WSSV) [14], consistent with its role as a conventional inhibitor. These observations implicate *SOCS2* in host-virus interactions across species, but whether it exerts broader regulatory function besides its canonical role remains an open question.

In silkworms, the JAK-STAT pathway operates in host defense against the *Bombyx mori* nucleopolyhedrovirus (BmNPV) [15,16,17,18,19]. The recent identification of an upstream interferon-like cytokine [20] and downstream homologs of interferon-stimulated genes (ISGs) [21,22] firmly establishes an evolutionarily conserved antiviral JAK-STAT axis in silkworms. *SOCS2* negatively regulates antibacterial immunity in silkworms by inhibition of the JAK-STAT pathway [23,24,25], but its role during viral infection is ambiguous. Although reduced *SOCS2* expression following STAT knockdown fits a linear feedback model [25], other observations suggest greater complexity. For example, Hsp90 inhibition increases STAT yet decreases *SOCS2* [26]. Moreover, upregulated *SOCS2* correlates with BmNPV resistance [27], contrary to expected immunosuppression. These discrepancies suggest that *SOCS2* functions beyond simple feedback inhibition.

Whether SOCS proteins can transcriptionally regulate STATs or other SOCSs has rarely been systematically tested, representing a conceptual gap in understanding the potentially reciprocal regulation within the SOCS-STAT network. We previously reported that silkworm *SOCS2* isoforms (*SOCS2L* and *SOCS2S*), along with *SOCS6/7*, are transcriptionally responsive to BmNPV infection, with *SOCS2L*/S showing the strongest induction [28,29,30]. Here, we investigate two key questions in the widely used BmN cell model: whether silkworm *SOCS2* isoforms promote BmNPV replication, and whether they transcriptionally regulate the SOCS-STAT network. Addressing these questions will clarify the roles of *SOCS2* in regulating the network and in viral infection, providing a foundation for further exploration of the network regulation and its role in silkworm-BmNPV interactions.

## 2. Materials and Methods

### 2.1. Cells and Virus

The silkworm BmN cell line and a recombinant BmNPV expressing red fluorescent protein (RFP-BmNPV, BV) were maintained in our laboratory. BmN cells were cultured at 27 °C in TC-100 medium (AppliChem Panreac, Darmstadt, Germany) supplemented with 10% fetal bovine serum (Gibco, NY, USA) and antibiotics.

### 2.2. Plasmid Construction

The full-length coding sequences for SOCS2S (GenBank: NM_001256992.1) and SOCS2L (GenBank: XM_038021379.1) proteins were cloned into the pIB/V5-His vector (Invitrogen, CA, USA) with an N-terminal EGFP tag. To minimize potential interference with *SOCS2* function, the tag was placed at the N-terminus, as the C-terminal SOCS box is critical for protein-protein interactions, whereas the N-terminal region consists primarily of flexible loops [31,32].

SH2 domain point mutations (R123Q for *SOCS2S*; R142Q for *SOCS2L*) were introduced by overlap extension PCR. This arginine residue in the SH2 domain is highly conserved across species and essential for phosphotyrosine interaction. Mutations targeting this residue have been well characterized to abolish phosphotyrosine-dependent substrate binding, thereby abrogating *SOCS2*-mediated JAK-STAT inhibition [31]. Given the high conservation between silkworm and human SH2 domains [28,29,30], these mutations are expected to similarly impair silkworm *SOCS2* function, allowing assessment of its role in viral infection. The experimental validation of these silkworm *SOCS2* mutants on substrate binding and JAK-STAT activity will be addressed in future work exploring mechanistic details. All constructs were verified by DNA sequencing. Primers are listed in Supplementary Table S1.

### 2.3. Transfection and Viral Infection

For transfection, the constructs included pIB-EGFP-SOCS2S/L and their SH2 domain mutants, with the empty pIB vector and pIB-EGFP serving as controls. Plasmids were purified using an Endo-Free BAC/PAC DNA Kit (Omega, Georgia, USA). BmN cells seeded in 24-well plates (~5 × 10^5^ cells/well) were transfected with 1 μg (low dose) or 10 μg (high dose) of plasmid DNA using Cellfectin II (Thermo Fisher Scientific, NY, USA). The two different doses were used in order to assess whether *SOCS2* regulates viral replication in a dose-dependent manner. For each well, a transfection mixture (200 μL) was prepared by combining Solution A (2 μL Cellfectin II in 98 μL serum-free medium) and Solution B (plasmid DNA in 100 μL serum-free medium). Cells were incubated with the mixture for 6 h, then the medium was replaced with fresh complete medium, and cells were cultured for an additional 48 h to allow *SOCS2* expression.

At 48 h post-transfection, cells were infected with RFP-BmNPV with a MOI of 5. Samples were collected at 0, 6, 12, and 24 h post-infection (hpi) for subsequent analyses. To assess viral gene expression, total RNA was extracted and subjected to quantitative real-time PCR (qPCR) to measure transcripts of the viral immediate-early gene *IE1* and the late capsid gene *VP39*. For viral DNA quantification, viral DNA was extracted from intracellular, extracellular, and total fractions, and quantified by qPCR using the *GP41* gene as a marker, with a standard curve generated from bacmid DNA. EGFP (from EGFP-*SOCS2* fusions) and RFP (viral marker) fluorescence were monitored using an inverted fluorescence microscope (Olympus, Tokyo Japan). Fluorescence intensities were quantified with ImageJ 1.54 software. Infectious virus titers in culture supernatants were determined by the TCID_50_ (50% tissue culture infectious dose) endpoint dilution assay. Briefly, BmN cells seeded in 96-well plates were inoculated with 10-fold serial dilutions (10^−1^ to 10^−8^) of culture supernatant. As preliminary experiments revealed that no new fluorescent wells emerged after day 5 post-infection (5 dpi) and the pattern remained stable, wells were scored for RFP fluorescence daily from 2 to 6 dpi with data on 6 dpi for calculation. TCID_50_ values were calculated using the commonly employed Reed-Muench method [33].

### 2.4. RNA Extraction and qPCR

RNA was extracted using TRIzol (Invitrogen, CA, USA) and reverse-transcribed using the HiScript III cDNA Synthesis Kit (Vazyme Biotech, Nanjing, China). qPCR was performed with AceQ SYBR Green Master Mix (Vazyme Biotech, Nanjing, China) on a QuantStudio 5 system (Thermo Fisher Scientific, NY, USA). The PCR cycling conditions comprised an initial denaturation at 95 °C for 30 s, followed by 39 cycles of 95 °C for 15 s, 58 °C for 30 s, and 72 °C for 30 s, with a subsequent melt curve analysis (60–95 °C, increment of 0.5 °C every 10 s). Gene-specific primers were designed using Primer 5.0 software and are listed in Supplementary Table S1. Relative expression levels were calculated using the 2^−ΔΔCt^ method, using α-tubulin as an internal control for qPCR and the reference gene for data normalization (GenBank: XM_021348632.1).

### 2.5. RNA Interference

Double-stranded RNAs (dsRNAs) targeting *SOCS2S* or *SOCS2L* (~500 bp) were synthesized using the TranscriptAid T7 High-Yield Transcription Kit (Thermo Fisher Scientific, NY, USA) from PCR templates flanked by T7 promoter sequence. After purification via chloroform extraction and ethanol precipitation, dsRNAs were resuspended in DEPC-treated water. dsRNAs targeting GFP (dsGFP) were synthesized as a negative control. For RNAi-mediated knockdown, BmN cells were transfected with 10 μg of dsRNA. Knockdown efficiency was assessed by qPCR at 48 h post-transfection. The impact of *SOCS2* knockdown on viral infection was analyzed as described above.

### 2.6. Statistical Analysis

All experiments included at least three technical replicates and were repeated independently three times. Data are presented as mean ± SD from three independent experiments (*n* = 3). Statistical significance for comparisons between two groups was evaluated by Student’s *t*-test. For comparisons involving multiple groups, time points, or doses, two-way ANOVA followed by Tukey’s post-hoc test was performed using GraphPad Prism 9.0. Significance levels were indicated as follows: *, *p* < 0.05; **, *p* < 0.01; ***, *p* < 0.001.

## 3. Results

### 3.1. Expression of SOCS2 in BmN Cells

**Figure 1:**
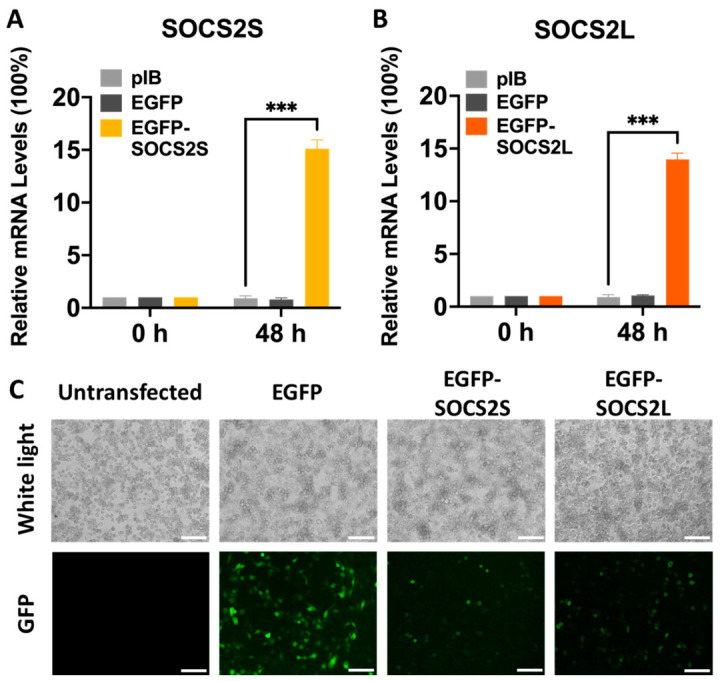
Expression of *SOCS2* isoforms in BmN cells. The insect expression vector pIB/V5 was used to express EGFP alone or EGFP fused with *SOCS2* isoforms, using 10 μg of plasmid DNA for each transfection. (**A**,**B**), qPCR analysis at 48 h post transfection (hpt), respectively. (**C**), Fluorescence microscopy. Images were taken 48 hpt. Green fluorescence, EGFP alone or EGFP-tagged proteins. White light, cell morphology. Scale bar: 100 μm. Data are presented as mean ± SD from three independent experiments (*n* = 3). Statistical significance was determined using two-way ANOVA followed by Tukey’s post-hoc test, with significance levels indicated as *** *p* < 0.001.

To investigate isoform-specific functions, we generated N-terminal EGFP fusions of *SOCS2S* and *SOCS2L* (pIB-EGFP-*SOCS2S/L*), which minimizes structural disruption. qPCR confirmed ~15-fold overexpression of both isoforms at 48 h post-transfection relative to empty vector or EGFP-only controls (Figure 1A,B). Fluorescence microscopy verified protein expression, with green signal detected in both EGFP-fusion groups (Figure 1C). The lower fluorescence intensity of fusion proteins compared to EGFP alone may indicate rapid protein turnover, warranting future investigation.

### 3.2. Overexpression of SOCS2 Promotes BmNPV Proliferation

**Figure 2:**
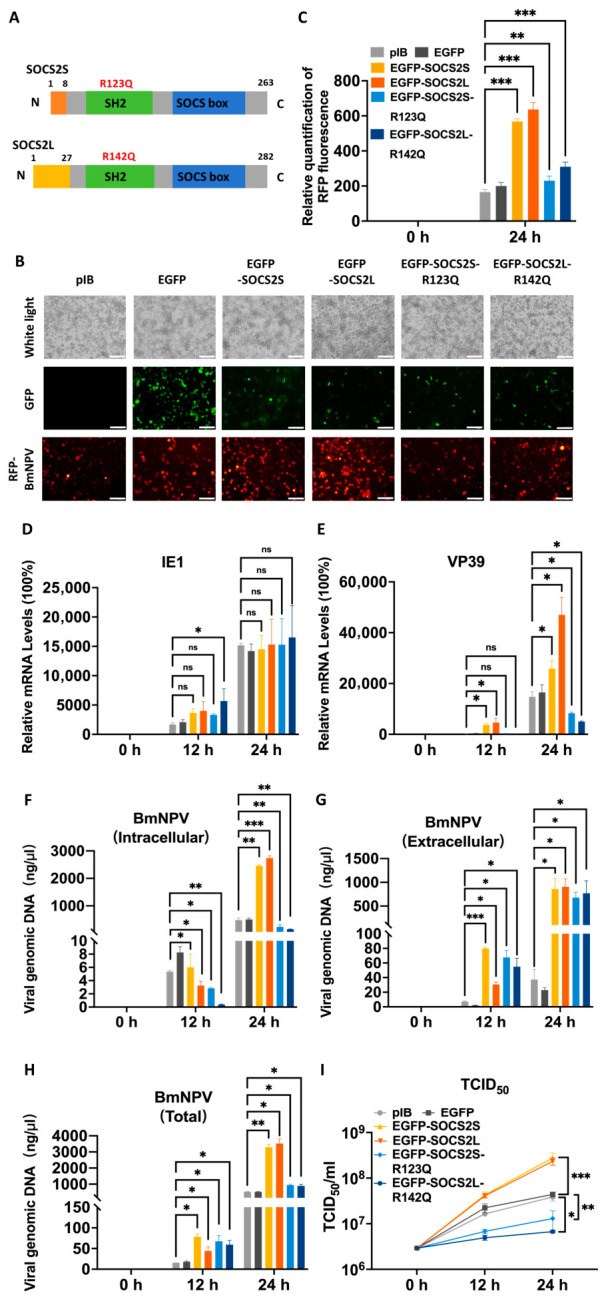
*SOCS2* overexpression promotes BmNPV proliferation. At 48 hpt of *SOCS2* expression plasmids, cells were infected with BmNPV (RFP, BV) and samples were taken 0, 12, 24 h post infection (hpi) for further analysis. (**A**), Mutation sites within the SH2 domain. R123Q for *SOCS2S* and R142Q for *SOCS2L*. (**B**), Fluorescence microscopy. Images were acquired 24 hpi. White light, cell morphology. Green fluorescence, EGFP alone or EGFP-tagged proteins. Red fluorescence, BmNPV replication. Scale bar: 100 μm. (**C**), Quantitative fluorescence analysis of BmNPV replication. The ImageJ 1.54 was used to count the red fluorescence in (**B**). (**D**,**E**), qPCR analysis of viral gene expression, D for *IE1* and E for *VP39*. (**F**–**H**), Quantification of viral genome replication. Intracellular (**F**), extracellular (**G**), and total (**H**) viral genomic DNA levels were analyzed by qPCR using *GP41* gene as a marker. (**I**), Viral titer determination. The culture supernatant was harvested, serially diluted and used to infect fresh cells to calculate the viral titers (TCID_50_ mL^−1^) based on red fluorescence observation. Data are presented as mean ± SD from three independent experiments (*n* = 3). Statistical significance for all panels was determined using two-way ANOVA followed by Tukey’s post-hoc test, with significance levels indicated as *** *p* < 0.001, ** *p* < 0.01, * *p* < 0.05, and ns (not significant, *p* > 0.05).

To assess the effect of *SOCS2* overexpression on BmNPV infection, BmN cells overexpressing *SOCS2L* or *SOCS2S* were infected with RFP-BmNPV; viral replication was monitored via fluorescence microscopy, viral gene transcription was assessed via qPCR of *IE1* and *VP39*, viral DNA replication was determined via qPCR of *GP41*, and infectious progeny production was ascertained via TCID_50_. Figure 2A shows the domain structures of SOCS2L and SOCS2S proteins.

At 24 hpi, RFP fluorescence was markedly higher in *SOCS2L/S*-expressing cells than controls (Figure 2B,C), indicating enhanced viral propagation. Both isoforms significantly increased viral DNA levels, with temporal differences. By 12 hpi, total and extracellular viral DNA (tvDNA, evDNA) were elevated while intracellular viral DNA (ivDNA) remained comparable to controls. By 24 hpi, all fractions, particularly evDNA, were substantially increased, suggesting accelerated viral genome amplification and release (Figure 2F–H). Transcriptional analysis revealed strong upregulation of the late capsid gene *VP39* at both time points (Figure 2E), whereas the immediate-early gene *IE1* was only marginally affected (Figure 2D). Consistent with these findings, TCID_50_ assays confirmed significantly higher infectious progeny titers in supernatants of *SOCS2L/S*-overexpressing cells (Figure 2I). Notably, *SOCS2L* exhibited greater proviral potency than *SOCS2S*, with stronger *VP39* upregulation at 24 hpi. Conversely, *SOCS2S* showed more pronounced early effects on viral DNA replication and release at 12 hpi, indicating a temporal division of labor between the two isoforms.

### 3.3. SOCS2 Promotes BmNPV Proliferation via SH2 Domain-Dependent and -Independent Mechanisms

To verify the proviral function of *SOCS2* isoforms, we introduced loss-of-function mutations (R123Q in *SOCS2S*; R142Q in *SOCS2L*) into SH2 domains of their proteins (Figure 2A). At 24 hpi, viral RFP fluorescence in mutant-expressing cells was marginally above controls but significantly below wild-type (WT) levels (Figure 2B,C), indicating these mutations attenuated the enhancement of viral proliferation by WT *SOCS2* isoforms. Notably, at 12 hpi, the tvDNA in mutant groups matched WT and exceeded controls; by 24 hpi, it fell below WT and only slightly above controls (Figure 2H). The ivDNA in mutant groups remained below WT and controls at all time points (Figure 2F). These results suggest that an intact SH2 domain is important for the enhancement of viral DNA replication by *SOCS2* isoforms.

Remarkably, the evDNA in the mutant groups remained at the high levels, comparable to WT levels and consistently exceeding control levels (Figure 2G), suggesting an SH2-independent role for *SOCS2* isoforms in promoting viral DNA release. To quantitatively assess the release, we calculated the extracellular-to-intracellular viral DNA ratio (eivDNA-ratio) as a measure of release efficiency. At 12 hpi, the eivDNA-ratios reached ~23.6 (*SOCS2S* mutant) and ~142.6 (*SOCS2L* mutant), compared with ~13.9–9.5 for their respective WT groups and ~1.9–1.0 for controls (vector and EGFP alone). These results indicate that both WT and mutant *SOCS2* isoforms enhance viral DNA release, with mutants exhibiting greater efficiency than WT counterparts, and *SOCS2L* demonstrating higher efficiency than *SOCS2S*. By 24 hpi, the ratios declined to ~3.0 (*SOCS2S* mutant) and ~5.1 (*SOCS2L* mutant); WT and control ratios also decreased to ~0.3 and ~0.1, respectively, likely due to late-stage intracellular accumulation of viral DNA. Nevertheless, the differential release efficiency remained consistent and evident. Collectively, relative to controls, overexpression of WT *SOCS2S* and *SOCS2L* markedly promoted viral DNA release, and overexpression of their SH2 mutants elicited even more pronounced effects.

Transcriptional analysis showed that SH2 mutants, like WT, minimally affected *IE1* (Figure 2D). However, they failed to upregulate *VP39* as WT did, and instead drove *VP39* expression below control levels (Figure 2E). Accordingly, TCID_50_ assays revealed severe reduction in infectious progeny titers in mutant groups, below both WT and controls (Figure 2I). Thus, *SOCS2* isoforms promote the release of viral DNA, likely as intact virions, given the concomitant increase in infectious titers and viral DNA. In contrast, the SH2 mutation does not simply abrogate function; rather, it differentially affects viral DNA replication, particle assembly, and release, uncoupling DNA replication from assembly/release. Moreover, the SH2 mutation enhances the export of viral DNA, likely in the form of non-infectious viral DNA or defective particles, as evidenced by a sharp reduction in infectious titers and *VP39* expression, despite WT levels of evDNA. It should be noted that the nature of these evDNA species was not experimentally determined, a limitation of the present study.

Together, our data indicate that an intact SH2 domain is required for the multiple pro-viral functions of silkworm *SOCS2* isoforms. Moreover, the SH2 mutation appears to convert *SOCS2* from an activator to an inhibitor of *VP39* expression, which may lead to damaged virion assembly and thus reduced viral titers. Intriguingly, the release of viral DNA/particles is promoted independently of the SH2 domain, an effect even amplified upon mutation. *SOCS2L* mutant exhibited stronger inhibition than *SOCS2S* mutant, consistent with isoform-specific regulatory potency.

### 3.4. SOCS2 Promotes BmNPV Replication in a Dose-Dependent Manner

**Figure 3:**
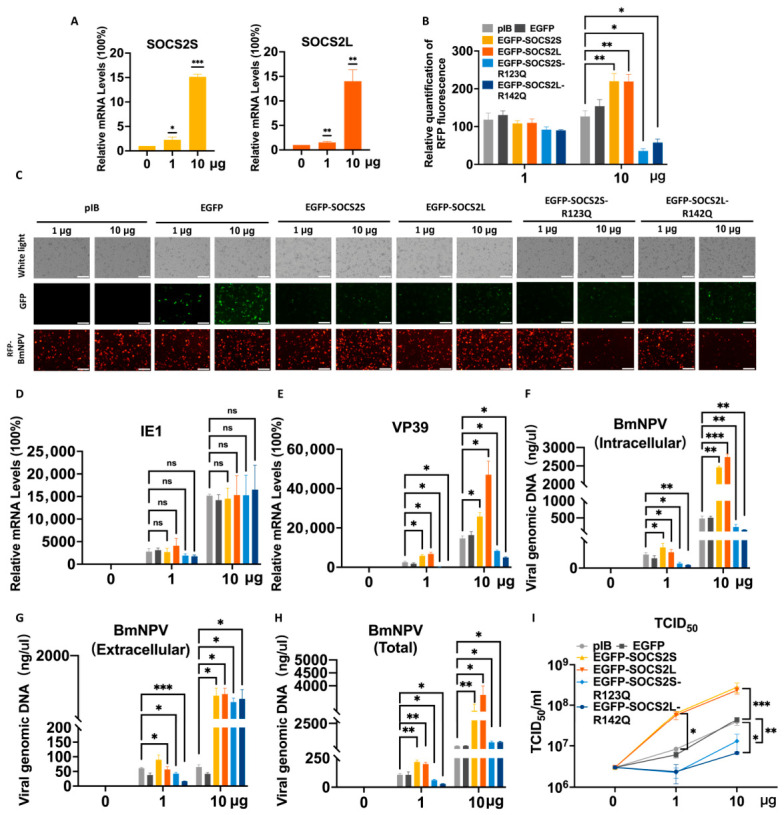
*SOCS2* promotes BmNPV proliferation in a dose-dependent manner. At 48 hpt of different doses of *SOCS2* plasmids (1 μg and 10 μg), cells were infected with BmNPV (RFP, BV) and samples were taken 24 hpi for further analysis. (**A**), qPCR validation of *SOCS2S* and *SOCS2L* overexpression. (**B**), Quantification of fluorescence for BmNPV replication. The ImageJ 1.54 was used to count the red fluorescence in (**C**). (**C**), Fluorescence microscopy. White light, cell morphology. Green fluorescence, EGFP alone or EGFP-tagged proteins. Red fluorescence, BmNPV-RFP. Scale bar: 100 μm. (**D**,**E**), qPCR analysis of viral gene expression, D for *IE1* and E for *VP39*. (**F**,**H**), Viral genome replication analysis. Intracellular (**F**), extracellular (**G**), and total (**H**) viral genomic DNA were isolated and quantified by qPCR, using *GP41* gene as a marker. (**I**), Viral titer determination. Culture supernatant was harvested, serially diluted and used to infect fresh cells to calculate the viral titers (TCID_50_ mL^−1^) based on red fluorescence observation. Data are presented as mean ± SD from three independent experiments (*n* = 3). Statistical significance was determined using Student’s *t*-test for (**A**), and two-way ANOVA followed by Tukey’s post-hoc test for (**B**), (**D**–**I**), with significance levels indicated as *** *p* < 0.001, ** *p* < 0.01, * *p* < 0.05, and ns (not significant, *p* > 0.05).

To further confirm the proviral function of *SOCS2* isoforms, we transfected BmN cells with low (1 µg) or high (10 µg) doses of expression plasmids. qPCR confirmed dose-dependent overexpression at 48 h post-transfection (Figure 3A). The fusion protein fluorescence (green) was markedly higher at the high dose than the low dose, providing a clear protein gradient (Figure 3B). Viral RFP fluorescence analysis revealed that high-dose overexpression of wild-type *SOCS2L/S* enhanced viral replication, whereas SH2 mutants caused pronounced inhibition (Figure 3B,C). These differences were not readily observable at the low dose, confirming the utility of the high-dose condition.

Consistently, high-dose WT *SOCS2L/S* significantly increased all viral DNA fractions relative to low-dose conditions (Figure 3F–H). WT *SOCS2L/S* induced dose-dependent increases in tvDNA and ivDNA (22- to 36-fold and 42- to 61-fold, respectively), far exceeding controls. In contrast, mutant *SOCS2L/S* enhanced evDNA in a dose-responsive manner (16-fold for *SOCS2S* mutant; 45-fold for *SOCS2L* mutant) but suppressed ivDNA accumulation, yielding tvDNA levels substantially below WT *SOCS2L/S*. Notably, the eivDNA-ratio for the vector control declined from ~1.6 (low dose) to ~0.1 (high dose), EGFP control from ~2.9 to ~0.1, WT *SOCS2S* from ~1.5 to ~0.3, and WT *SOCS2L* from ~1.3 to ~0.3. These decreases were largely attributable to intracellular accumulation of viral DNA at the high dose. However, the ratio for *SOCS2L* mutant increased from ~1.8 at the low dose to ~5.1 at the high dose, whereas the ratio for *SOCS2S* mutant remained comparably elevated at both doses (~3.3 and ~3.0, respectively). Compared with controls, WT *SOCS2* isoforms exhibited increased release efficiency only at the high dose, whereas the mutants, particularly *SOCS2L*, displayed enhanced release efficiency at both doses, with a substantially greater increase under high-dose conditions relative to both controls and WT isoforms. Together, these results unmask a dose-responsive, SH2-independent function of *SOCS2* that promotes the release of viral DNA/particles (whether mature or immature).

Notably, both WT and mutant *SOCS2L/S* dose-dependently upregulated *IE1* and *VP39* relative to their own low-dose conditions, but neither significantly altered *IE1* compared to controls (Figure 3D,E). In contrast, WT *SOCS2L/S* strongly upregulated *VP39*, whereas mutants largely lost this capacity. Accordingly, TCID_50_ assays showed that WT *SOCS2L*/S increased infectious titers dose-dependently, while mutants suppressed titers below both control and WT levels (Figure 3I). *SOCS2L* consistently exhibited stronger effects than *SOCS2S* in both promoting (WT) and suppressing (mutant) viral replication.

In summary, *SOCS2* isoforms promote BmNPV replication in a dose-dependent manner. SH2 domain mutation dissociates these functions: it abrogates dose-dependent enhancement of viral DNA replication and late gene expression while preserving (*SOCS2S*) and even augmenting (*SOCS2L*) the dose-responsive release of viral DNA/defective particles. *SOCS2L* exerts stronger regulatory potency than *SOCS2S*, underscoring isoform specialization.

### 3.5. RNAi-Mediated Silencing of SOCS2 Attenuates BmNPV Proliferation

**Figure 4:**
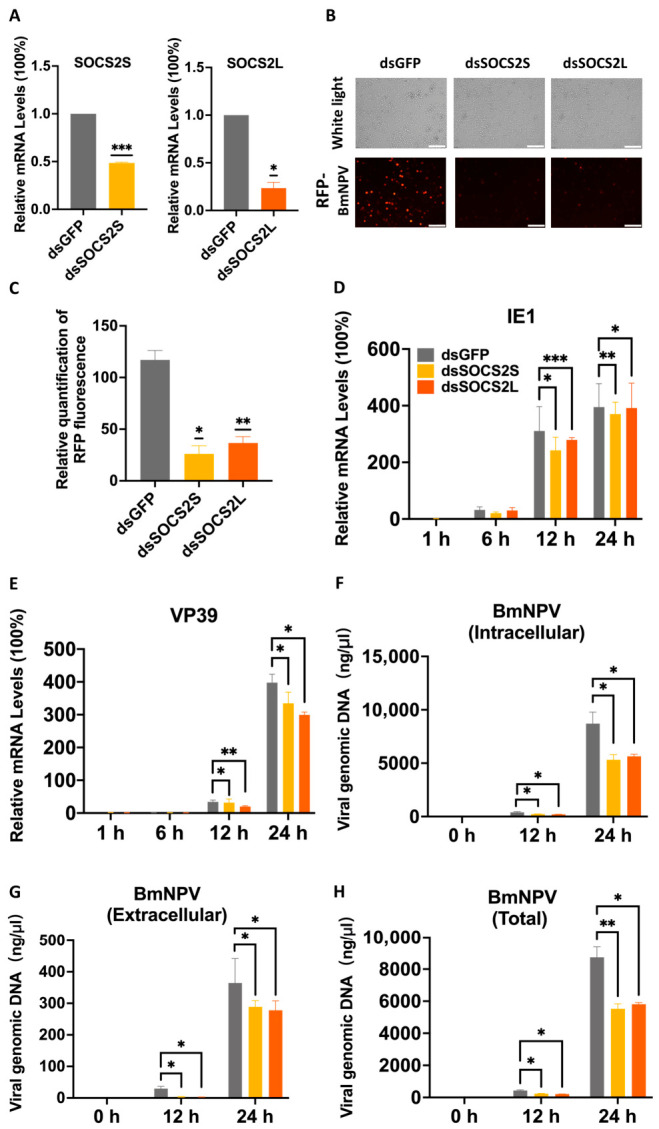
*SOCS2* knockdown attenuates BmNPV proliferation. BmN cells were transfected with dsRNAs for 48 h, samples taken for efficiency measurement. Then cells were infected with BmNPV-RFP and samples were taken at 0, 12, and 24 hpi for qPCR analysis. dsGFP was used as a control. (**A**), Knockdown efficiency verification for *SOCS2S* and *SOCS2L*. (**B**), Fluorescence microscopy at 24 hpi. White light, cell morphology. Red fluorescence, BmNPV-RFP. Scale bar: 100 μm. (**C**), Quantification of fluorescence for BmNPV replication. The ImageJ 1.54 was used to count the red fluorescence in (**B**). (**D**,**E**), qPCR analysis of viral gene expression, D for *IE1* and E for *VP39*. (**F**–**H**), Viral DNA replication analysis. Intracellular (**F**), extracellular (**G**), and total (**H**) viral genomic DNA were isolated and quantified by qPCR, using *GP41* gene as a marker. Data are presented as mean ± SD from three independent experiments (*n* = 3). Statistical significance was determined using Student’s *t*-test for (**A**,**C**), and two-way ANOVA followed by Tukey’s post-hoc test for (**D**–**H**), with significance levels indicated as *** *p* < 0.001, ** *p* < 0.01, * *p* < 0.05.

To validate the proviral role of *SOCS2* isoforms, we knocked down *SOCS2L* and *SOCS2S* using sequence-specific dsRNAs, with dsGFP as a control. qPCR confirmed efficient knockdown at 48 h post-transfection, reducing *SOCS2S* and *SOCS2L* mRNA levels by 54% and 77%, respectively (Figure 4A).

Upon RFP-BmNPV infection, fluorescence microscopy revealed markedly reduced viral red fluorescence in both knockdown (KD) groups at 24 hpi (Figure 4B,C), indicating impaired viral propagation. This reduction was corroborated at the molecular level: *SOCS2L*-KD and *SOCS2S*-KD modestly decreased *IE1* transcription (Figure 4D) but more strongly suppressed *VP39* (24% for *SOCS2S*; 30% for *SOCS2L*) (Figure 4E). Viral genome replication was also significantly impaired, with tvDNA, ivDNA, evDNA levels all markedly lower in these knockdown cells (Figure 4F–H).

Notably, at 12 hpi, the eivDNA-ratio was ~0.07 for the dsGFP control group, ~0.02 for the *SOCS2S*-KD group, and ~0.01 for the *SOCS2L*-KD group, indicating knockdown of *SOCS2* isoform reduces the efficiency of viral DNA release. This finding is consistent with the overexpression experiments. However, by 24 hpi, the ratios across all groups converged to comparable levels (~0.04, 0.05, and 0.05, respectively), the reasons of which remain unknown.

Collectively, depletion of either *SOCS2* isoform attenuates BmNPV replication by inhibiting viral genome replication, late gene expression and viral DNA release, further confirming *SOCS2L/S* as proviral host factors.

### 3.6. SOCS2 Modulates the mRNA Expression of SOCS-STAT Network Members

To determine whether *SOCS2* isoforms transcriptionally modulate the SOCS-STAT network, we profiled mRNA levels of network components following overexpression or knockdown of *SOCS2L* or *SOCS2S* under virus-free conditions. The comparison was based on steady-state mRNA levels at 48 h relative to 0 h in each setting. The functional consequences of these changes for JAK-STAT pathway remain to be determined in future studies. Low-dose overexpression (LD-OE) and high-dose overexpression (HD-OE) were defined as described.

**Figure 5:**
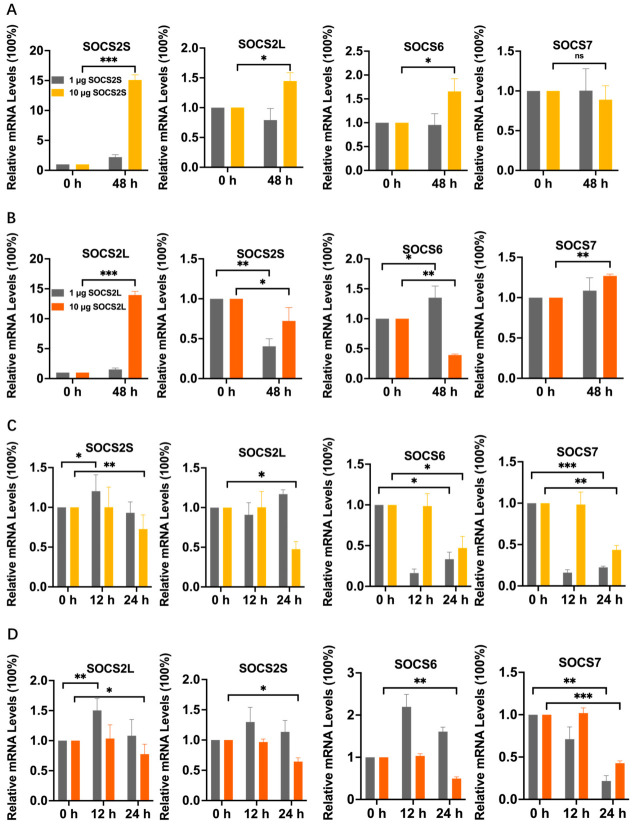
*SOCS2* overexpression and BmNPV infection cooperatively regulate the mRNA expression of SOCS family members. qPCR was used to measure the effects of overexpression of *SOCS2L* and *SOCS2S* with different doses (1 μg or 10 μg) on SOCS family genes with or without BmNPV infection in BmN cells. (**A**,**B**), Overexpression of *SOCS2S* (**A**) or *SOCS2L* (**B**) without viral infection. (**C**,**D**), Overexpression of *SOCS2S* (**C**) or *SOCS2L* (**D**) with viral infection. Data are presented as mean ± SD from three independent experiments (*n* = 3). Statistical significance for all panels was determined using two-way ANOVA followed by Tukey’s post-hoc test, with significance levels indicated as *** *p* < 0.001, ** *p* < 0.01, * *p* < 0.05, and ns (not significant, *p* > 0.05).

**Figure 6:**
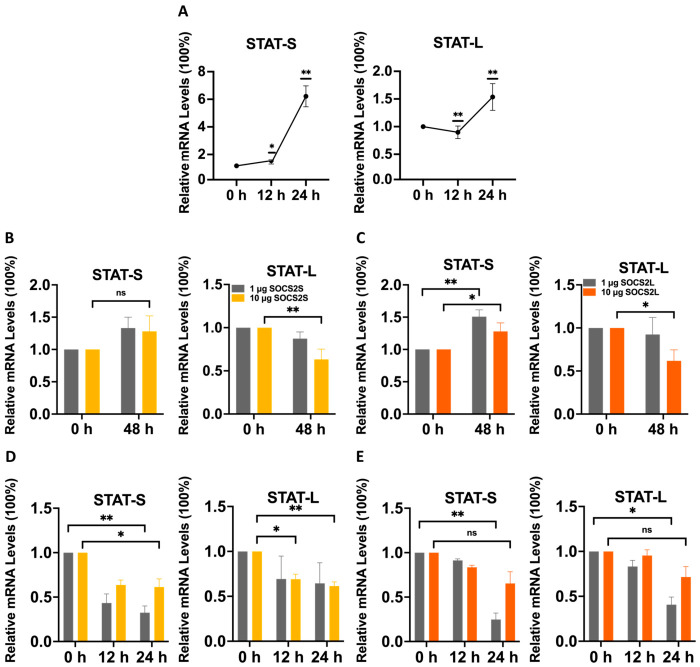
*SOCS2* overexpression and BmNPV infection cooperatively regulate the mRNA expression of STAT family members. qPCR was used to measure the effects of overexpression of *SOCS2L* and *SOCS2S* with different doses (1 μg or 10 μg) on STAT family genes (*STAT-L/S*) with or without BmNPV infection in BmN cells. (**A**), *STAT-L/S* upregulation by BmNPV infection. (**B**,**C**), Overexpression of *SOCS2S* (**B**) or *SOCS2L* (**C**) without viral infection. (**D**,**E**) Overexpression of *SOCS2S* (**D**) or *SOCS2L* (**E**) with viral infection. Data are presented as mean ± SD from three independent experiments (*n* = 3). Statistical significance for all panels was determined using two-way ANOVA followed by Tukey’s post-hoc test, with significance levels indicated as ** *p* < 0.01, * *p* < 0.05, and ns (not significant, *p* > 0.05).

**Figure 7:**
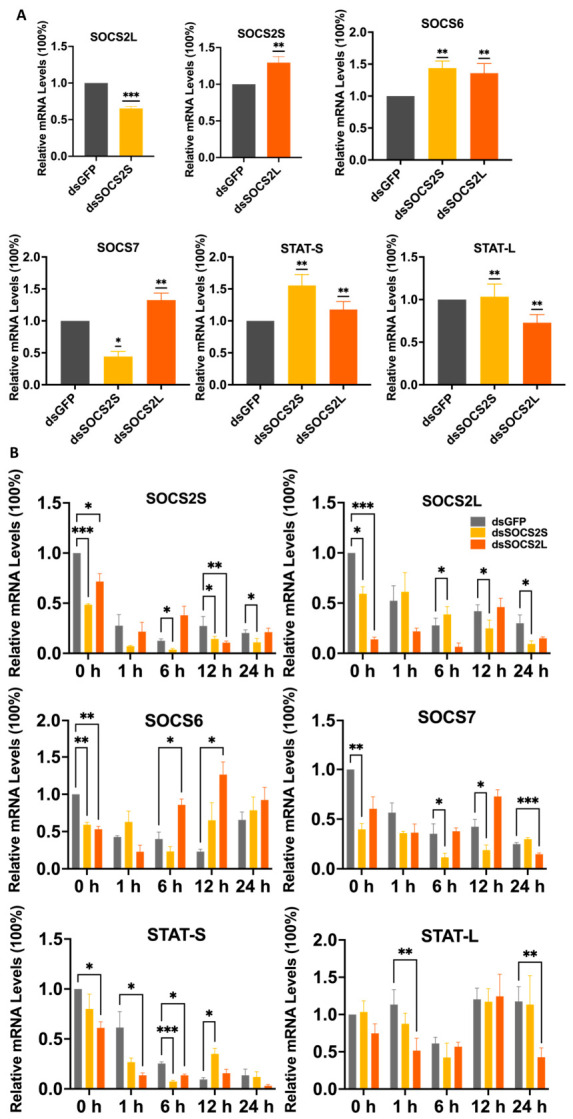
*SOCS2* knockdown and BmNPV infection cooperatively regulate the mRNA expression of SOCS-STAT network members. BmN cells were transfected with dsRNAs for 48 h, samples taken for qPCR assay. Then cells were infected with BmNPV-RFP and samples were taken at 0, 12, and 24 hpi for qPCR analysis. dsGFP was used as a control. (**A**) Effects of *SOCS2* knockdown without viral infection. (**B**) Effects of *SOCS2* knockdown with viral infection. Data are presented as mean ± SD from three independent experiments (*n* = 3). Statistical significance was determined using Student’s *t*-test for (**A**), and two-way ANOVA followed by Tukey’s post-hoc test for (**B**), with significance levels indicated as *** *p* < 0.001, ** *p* < 0.01, * *p* < 0.05.

Modulating *SOCS2* isoforms altered mRNA levels of SOCS-STAT network components in a dose- and isoform-dependent manner. Under LD-OE, *SOCS2L* strongly downregulated *SOCS2S* (~60%) and upregulated *SOCS6* (~40%), whereas *SOCS2S* mildly downregulated *SOCS2L* (~20%) and *SOCS6* (<10%), with neither isoform affecting *SOCS7*. Under HD-OE, *SOCS2L* downregulated *SOCS2S* (~30%) and *SOCS6* (~60%) but upregulated *SOCS7* (~30%), whereas *SOCS2S* upregulated *SOCS2L* (~40%) and *SOCS6* (~70%) but mildly downregulated *SOCS7* (Figure 5A,B). Both isoforms increased *STAT-S* (~30–50%) and slightly decreased *STAT-L* (~10%) under LD-OE, but they comparably increased *STAT-S* (~30%) and decreased *STAT-L* (~40%) under HD-OE (Figure 6B,C). For knockdown, *SOCS2S*-KD downregulated *SOCS2L* (~35%) and *SOCS7* (~57%) but upregulated *SOCS6* (~43%), and increased *STAT-S* (~54%) without affecting *STAT-L*. In contrast, *SOCS2L*-KD upregulated *SOCS2S*, *SOCS6*, and *SOCS7* by ~30–35% each, and modestly increased *STAT-S* (~17%) but markedly decreased *STAT-L* (~30%) (Figure 7A).

Collectively, these data demonstrate that modulation of *SOCS2L/S* expression alters mRNA levels of the SOCS-STAT network in a dose-, isoform-, and target-dependent manner, indicating that *SOCS2L/S* may serve as a regulatory node capable of reshaping the SOCS-STAT transcriptional landscape. Notably, *STAT-S* was consistently upregulated while *STAT-L* was generally suppressed, providing the first evidence that STAT members can be differentially regulated by a single SOCS family member.

### 3.7. SOCS2 and BmNPV Cooperatively Alter the mRNA Levels of SOCS-STAT Network Members

We previously reported that BmNPV upregulates *SOCS2S*, *SOCS2L*, and *SOCS7* [28,30] but downregulates *SOCS6* [29] at the mRNA levels. Here, we found that BmNPV also upregulates mRNA levels of both *STAT-S* and *STAT-L* (Figure 6A). Given that *SOCS2* isoforms regulate mRNA levels of the SOCS-STAT network, we next investigated whether they cooperate with BmNPV to remodel this network at the mRNA level.

As shown in Figure 5C,D and Figure 6D,E, compared with overexpression alone (0 hpi), LD-OE of *SOCS2S* plus infection at 12 hpi upregulated *SOCS2S* (~20%), but downregulated *SOCS2L* (~10%), *SOCS6* (~80%), *SOCS7* (~80%), *STAT-L* (~30%), and *STAT-S* (~60%). In contrast, LD-OE of *SOCS2L* plus infection upregulated *SOCS2S* (~30%), *SOCS2L* (~50%), and *SOCS6* (120%), while downregulating *SOCS7* (~30%), *STAT-L* and *STAT-S* (~20% each). HD-OE of either isoform plus infection resulted in no significant changes in SOCS members, but HD-OE of *SOCS2L* decreased *STAT-S* (~20%) without affecting *STAT-L*, whereas HD-OE of *SOCS2S* decreased both STAT isoforms (~30–40%). By 24 hpi, LD-OE of *SOCS2S* plus infection downregulated *SOCS2S* (~10%), *SOCS6* (~70%), *SOCS7* (~80%), *STAT-L* (~40%) and *STAT-S* (~70%), but upregulated *SOCS2L* (~20%). In contrast, LD-OE of *SOCS2L* plus infection upregulated *SOCS2S* (~10%), *SOCS2L* (~10%), and *SOCS6* (~60%), while downregulating *SOCS7* (~80%) and both STAT isoforms (~60–70%). HD-OE of *SOCS2L* plus infection downregulated all SOCS members by ~20–60% and STAT isoforms by ~30% each, while HD-OE of *SOCS2S* plus infection downregulated all SOCS members by ~30–60% and STAT isoforms by ~40% each.

As shown in Figure 7B, the expression of *SOCS2S*, relative to baseline (infection onset, 0 hpi), exhibited a sharp decline followed by a partial recovery across all knockdown groups, with the most pronounced fluctuations observed in the *SOCS2L*-KD group. In contrast, *SOCS2L* expression in the dsGFP control group initially decreased and then increased, but remained stably downregulated throughout in the *SOCS2S*-KD group, whereas it rebounded sharply in the *SOCS2L*-KD group, reaching approximately 5-fold and 1-fold of baseline at 12 and 24 hpi, respectively. *SOCS6* expression in the dsGFP control group progressively declined before recovering to ~70% of baseline by 24 hpi, but decreased only at 6 hpi and subsequently exceeded baseline levels in the *SOCS2S*-KD group, while it dropped sharply at 1 hpi followed by continuous upregulation in the *SOCS2L*-KD group, reaching levels ~80%, ~160%, and ~80% above baseline at 6, 12, and 24 hpi, respectively. *SOCS7* expression in the dsGFP control group decreased until 6 hpi and then stabilized, but declined and subsequently recovered to ~75% of baseline by 24 hpi in the *SOCS2S*-KD group, while it increased by ~50% at 12 hpi before dropping to ~17% of baseline at 24 hpi in *SOCS2L*-KD Group. Notably, *STAT-S* expression in the dsGFP control group continuously decreased, reaching a minimum at 12 hpi; in the *SOCS2S*-KD group, it dropped to a minimum at 6 hpi, rebounded strongly at 12 hpi, and then declined again; in the *SOCS2L*-KD group, it plunged sharply at 1 hpi, remained low at 6 hpi, rebounded at 12 hpi, and then decreased sharply by 24 hpi. In contrast, *STAT-L* showed smaller fluctuations. In the dsGFP control group, *STAT-L* increased at most time points, except for a dip at 6 hpi. In both *SOCS2S*-KD and *SOCS2L*-KD groups, *STAT-L* initially decreased and then increased. Although *STAT-L* levels were comparable across groups at 12 hpi, its expression in the *SOCS2L*-KD group was significantly lower than in the other groups by 24 hpi.

Together, these results suggest that *SOCS2* isoforms cooperate with BmNPV to drive dynamic, context-dependent reorganization of the mRNA profiles of the SOCS-STAT network, distinct from either perturbation alone.

## 4. Discussion

This study provides the first systematic experimental evidence that silkworm *SOCS2* isoforms act as proviral factors for BmNPV, promoting multiple stages of the viral life cycle through both SH2-dependent and SH2-independent mechanisms. Furthermore, *SOCS2* and BmNPV individually and cooperatively modulate the mRNA expression of SOCS-STAT network members, extending the function of *SOCS2* from a simple feedback inhibitor to a potential transcriptional modulator. These findings underscore the significance of *SOCS2* within the SOCS-STAT network, suggesting it may be hijacked by viruses to evade host immunity.

### 4.1. SOCS2 Promotes BmNPV Replication at Multiple Stages with SH2 Domain-Dependent and -Independent Mechanisms

Silkworm *SOCS2* isoforms promote BmNPV replication at multiple stages of the viral life cycle. Specifically, they enhance viral DNA replication, late gene expression (*VP39*), infectious virus titers, and the release of viral DNA/mature or immature particles. These findings provide the first evidence that a SOCS family member can exert multiple proviral functions. Interestingly, *SOCS2* isoforms have only marginal effects on *IE1* expression, suggesting they may bypass it and act at downstream steps to facilitate viral replication. This may point to a novel virus-host interaction mechanism, wherein a host SOCS member promotes viral replication independently of viral immediate-early genes. We are currently performing a comprehensive analysis of the regulation of viral gene expression by silkworm *SOCS2* at both the mRNA and protein levels, which will likely provide deeper mechanistic understanding.

We employed a well-characterized SH2 domain loss-of-function mutation targeting the conserved arginine originally established in mammalian *SOCS2* to investigate the role of silkworm *SOCS2* in viral infection. The conservation of both the critical arginine and the entire SH2 domain in silkworm *SOCS2* [30] strongly supports that these mutations similarly disrupt SH2 function and abrogate *SOCS2*-mediated JAK-STAT inhibition, thereby allowing functional interrogation of *SOCS2* isoforms during viral infection. Indeed, the SH2 domain mutation of silkworm *SOCS2* isoforms reduced their enhancement of viral DNA replication to control levels. Moreover, the mutation not only diminished their enhancement of *VP39* expression and production of infectious progeny, but also reduced both to below control levels. These findings suggest the importance of an intact SH2 domain for the pro-viral function of silkworm *SOCS2* isoforms, which apparently exert differential effects on distinct stages of viral replication. Interestingly, high levels of extracellular viral DNA (comparable to WT levels) from mutant-expressing cells, coupled with very low infectivity, indicate that the released material consists predominantly of non-infectious viral DNA or defective particles, implying severely impaired virion assembly. Importantly, the suppression of *VP39* and the defects in virion assembly clearly exceed the reduction in viral DNA replication, demonstrating that these phenotypes are not merely downstream consequences of impaired genome amplification. The SH2 mutation apparently uncouples viral DNA replication from proper assembly and egress, suggesting the SH2 domain may also act as a quality-control checkpoint specifically required for late gene expression and proper virion assembly, a function distinct from its role in promoting DNA replication. Thus, silkworm *SOCS2* regulates multiple stages of the BmNPV life cycle via SH2-dependent yet mechanistically distinct pathways. The use of SH2 mutants reveals the critical requirement of this conserved domain for proviral functions and helps delineate stage-specific regulation, underscoring the value of such mutations.

Notably, both WT and SH2-mutant *SOCS2* isoforms promote the release of viral DNA/particles, a previously unrecognized function for *SOCS2*. Our data suggest that the extracellular viral DNA (evDNA) from WT groups originates predominantly from intact virions, whereas that from mutant groups likely consists primarily of non-infectious, defective particles or naked viral DNA. The precise nature of these evDNA species was not determined in the present study, which will be addressed in future work employing transmission electron microscopy (TEM), DNase protection assays, and Western blotting for the envelope protein GP64. Despite this limitation, SH2 mutants exhibit higher release efficiency than WT isoforms, indicating that the release-promoting function of *SOCS2* is independent of its SH2 domain. To our knowledge, this is the first report that *SOCS2* promotes viral DNA/particle release, implicating *SOCS2* in viral egress and transmission.

Direct SOCS-virus interactions remain rarely studied, with the exception of two reports on *SOCS1* and *SOCS3*. *SOCS1* binds HIV-1 Gag via its SH2 domain to enhance Gag stability and trafficking, independent of the essential arginine required for phosphotyrosine recognition [34]. *SOCS3* binds Ebola virus VP40 to promote its ubiquitination and virus budding, although the SH2 requirement remains unclear [35]. Although silkworm lacks true orthologs of *SOCS1* and *SOCS3*, other family members including *SOCS2* share a conserved domain architecture. Thus, we hypothesize that silkworm *SOCS2* may bind BmNPV proteins, such as *VP39* or *GP64*, to promote virion assembly and release, which will be tested using GST pull-down and Co-IP assays. Interestingly, BmNPV utilizes the multivesicular body (MVB) pathway to release both mature virions and naked nucleocapsids [36], offering a potential explanation for the phenotype of SH2 mutants. Upon SH2 mutation, the cell may redirect immature or defective particles to the MVB pathway for export. To test it, we will knock down key MVB genes, such as *VPS4*, to determine the effect on viral DNA release. Furthermore, we speculate the existence of a putative “release inhibitor” that ensures release of only properly assembled virions. WT *SOCS2* may recognize and degrade this inhibitor to facilitate release, whereas SH2 mutants may fail to degrade it, thereby suppressing release. Concurrently, accumulation of viral DNA and/or defective particles may trigger a “waste-export pathway” to expel these aberrant products. To test it, we will compare the interactomes of WT and SH2-mutant *SOCS2* using Co-IP coupled with mass spectrometry (MS) to identify candidate inhibitors, followed by functional validation.

### 4.2. SOCS2 Promotes BmNPV Proliferation in a Dose-Dependent Manner

To investigate the dose-dependent effects of *SOCS2*, we employed both low- and high-dose overexpression. This dose-gradient strategy proved critical for dissecting the multifaceted roles of *SOCS2* in BmNPV infection. Using this approach, we observed that WT *SOCS2* isoforms promote viral replication in a dose-dependent manner. High-dose WT *SOCS2* markedly enhanced multiple stages of viral life cycle, whereas low-dose effects were minimal. This gradient effect not only rules out non-specific overexpression artifacts but also establishes a direct, quantifiable causal relationship between *SOCS2* and viral replication, providing strong evidence for its proviral function. Furthermore, the threshold effect suggests that viruses may have evolved mechanisms to upregulate *SOCS2* expression, thereby hijacking its proviral activity during infection.

The dose-dependent strategy was particularly instrumental in uncovering the SH2-independent function of *SOCS2*. Low-dose SH2 mutants showed no significant phenotypes; however, at the high-dose, they exhibited a striking functional uncoupling of viral DNA replication and gene expression from viral DNA/defective particle release. Notably, the release efficiency for these mutants was higher than controls at the low-dose and was further substantially increased at the high-dose, while the efficiency for WTs was comparable to controls at the low-dose but was much higher than controls at the high-dose. These results demonstrate the value of using the high-dose, which unmasks a previously unrecognized, SH2-independent function of *SOCS2* that promotes viral DNA/particle release. This dose-dependent uncoupling indicates that *SOCS2* may actively regulate viral egress in an independent viral expulsion pathway, possibly involving vesicular transport or membrane remodeling.

In summary, the dose-dependent effects observed in this study not only validate the specificity of *SOCS2*-mediated proviral activity but also underscore the necessity of high-expression conditions to unmask latent functions, particularly the SH2-independent promotion of viral DNA release and the dominant-negative suppression of late gene expression by SH2 mutants. These findings establish dose titration as a critical experimental strategy for dissecting the multifaceted roles of rapid-turnover host proteins like *SOCS2* in viral infection. To determine the physiological relevance of the high-dose condition, we will examine whether endogenous *SOCS2* is induced to comparable levels during BmNPV infection in future studies.

### 4.3. SOCS2 Isoforms Modulate the mRNA Expression of SOCS-STAT Network Members in Isoform-, Target Gene- and Dose-Dependent Manner

Our data unveil that *SOCS2L* and *SOCS2S* transcriptionally regulate SOCS-STAT network in an isoform-specific, target-selective, and dose-dependent manner.

The overexpression and knockdown results suggest that under basal conditions, *SOCS2L* and *SOCS2S* may form a negative feedback loop with *SOCS2L* unidirectionally inhibiting *SOCS2S* while *SOCS2S* regulating *SOCS2L* in a bidirectional, dose-dependent manner. Upon overexpression, they oppositely regulate *SOCS6*, yet only *SOCS2L* regulates *SOCS7*. Knockdown data indicate that *SOCS2L* acts as a broad-spectrum inhibitor maintaining network homeostasis, while *SOCS2S* plays dual roles, positively regulating *SOCS2L* and *SOCS7*, yet inhibiting *SOCS6*.

Notably, regardless of overexpression or knockdown, *SOCS2L* and *SOCS2S* consistently upregulate *STAT-S* and downregulate *STAT-L* except for the weak effect of *SOCS2S* knockdown on *STAT-L*, indicating possibly a non-linear, indirect, or compensatory regulation rather than a simple negative feedback loop. These findings offer insights into how *SOCS2* selectively controls STAT isoforms to modulate JAK-STAT signaling and viral replication. Consistent with a recent report showing antiviral dominance of *STAT-L* over *STAT-S* [17], our work reveals an additional layer of transcriptional regulation imposed by *SOCS2* on functionally divergent STAT isoforms, underscoring its significance in antiviral defense.

In summary, under virus-free conditions, *SOCS2L* and *SOCS2S* may form an asymmetric regulatory node that maintains SOCS-STAT mRNA homeostasis through dose-dependent, isoform-specific mechanisms. This provides the first evidence that a SOCS member regulates the mRNA expression of other SOCS members and STATs, filling a long-standing knowledge gap in the field.

### 4.4. SOCS2 and BmNPV Cooperatively Modulate the mRNA Expression of SOCS-STAT Network Members

Our work reveals that BmNPV [28,29,30] and *SOCS2* isoforms modulate the SOCS-STAT mRNA landscape not only individually but also cooperatively, in a context-dependent manner that goes beyond simple additivity.

In overexpression settings, low-dose *SOCS2S* plus infection induced biphasic expression of *SOCS2L/S* with sustained suppression of *SOCS6/7*. Low-dose *SOCS2L* plus infection upregulated both isoforms, enhanced *SOCS6*, and persistently downregulated *SOCS7*. High-dose overexpression of either isoform plus infection had minor early effects but caused broad, late suppression of SOCS members. Both low- and high-dose overexpression suppressed *STAT-L* and reversed *STAT-S* from upregulation to downregulation with stronger effects at the high dose. This finding suggests a blockade of JAK-STAT antiviral signaling that promotes viral replication, consistent with our functional data. Despite lack of analysis of control groups (empty vector or EGFP with infection) to evaluate impacts of overexpression context on virus-mediated regulation, the altered transcription versus overexpression alone suggests viral interplay with *SOCS2* isoforms.

Upon knockdown plus infection, the control group (dsGFP plus infection) exhibited downregulation of SOCS members and *STAT-S* with fluctuation of *STAT-L,* differing from infected cells without dsRNA possibly due to the knockdown procedure itself. In *SOCS2*-knockdown groups, *SOCS6* initially decreased but eventually rose above both baseline (0 hpi) and control levels, indicating knockdown-induced upregulation overrides viral suppression. In contrast, *SOCS7* showed asymmetric, time-dependent changes, suggesting complex synergism. *SOCS2S* knockdown further downregulated both *SOCS2* isoforms, suggesting a synergy with the virus. *SOCS2L* knockdown plus infection caused a strong rebound of *SOCS2L* at 12 hpi, revealing the virus reverses knockdown effects. *STAT-S* became persistently and strongly downregulated, overriding its upregulation by knockdown alone. *STAT-L* exhibited time-dependent downregulation or upregulation, with a late decline occurring only upon *SOCS2L* knockdown.

Thus, *SOCS2* isoforms appear to act as potential regulatory nodes that may modulate the mRNA expression of the SOCS-STAT network members during viral infection. They cooperate with or antagonize the virus in a dose-, isoform-, and target gene-specific manner, altering the host transcriptional landscape and potentially determining the success or failure of viral replication. The two isoforms display clear functional asymmetry: loss of *SOCS2L* provokes more pronounced and broader network perturbations than loss of *SOCS2S*, suggesting a dominant role for *SOCS2L* in maintaining transcriptional homeostasis during infection.

### 4.5. The Functional Difference of Silkworm SOCS2 Isoforms

We have previously reported that *SOCS2* exists as two isoforms in many Lepidopteran insects, whereas most other species, including mammals, possess only a single isoform, *SOCS2S*, suggesting Lepidoptera-specific functions [30]. In the present study, our data reveal that *SOCS2L* promotes BmNPV replication more potently than *SOCS2S*, with a notably stronger upregulation of *VP39*. Interestingly, wild-type *SOCS2S* promotes viral DNA release more strongly than *SOCS2L*, whereas the *SOCS2L* mutant exerts a stronger effect than the *SOCS2S* mutant. At the mRNA level, *SOCS2L* and *SOCS2S* exhibit complex regulation between themselves and on other SOCS members, which becomes even more intricate and highly dynamic in the presence of viral infection. Under basal conditions, *SOCS2L* appears to act as a broad suppressor of SOCS family members, whereas *SOCS2S* displays dose-dependent positive and negative regulation.

The mechanistic basis for these differences remains unknown. The two *SOCS2* isoforms share the same SH2 domain and SOCS box domain, differing only in a short N-terminal region. We speculate that this small but distinct N-terminal region may provide isoform-specific protein–protein interaction interfaces, potentially engaging different host or viral proteins, thereby influencing their ability to promote viral replication or modulate the transcription of network members. To dissect the underlying mechanisms, we plan to systematically screen for interacting viral and host proteins using Co-IP coupled with MS, with particular emphasis on those differentially interacting with the two isoforms.

### 4.6. Study Limitations

First, the present study was performed using the BmN cell model, and the findings lack in vivo validation. BmN cells are a commonly used silkworm cell line suitable for mechanistic screening. To validate the current findings and further explore the mechanism of action of *SOCS2* isoforms in vivo, we have established a silkworm BmNPV infection model and will employ RNAi to functionally dissect the SOCS-STAT network, thereby verifying the roles of *SOCS2* isoforms at the whole-organism level.

Second, our analysis of the SOCS-STAT network is limited to steady-state mRNA levels. To determine whether the observed mRNA changes translate into functional alterations in JAK-STAT pathway activity, future studies will use western blotting and immunofluorescence to measure protein expression levels of pathway components, *STAT-L/S* phosphorylation, and downstream effector expression following *SOCS2* overexpression or knockdown, both in the presence and absence of viral infection.

## 5. Conclusions

**Figure 8:**
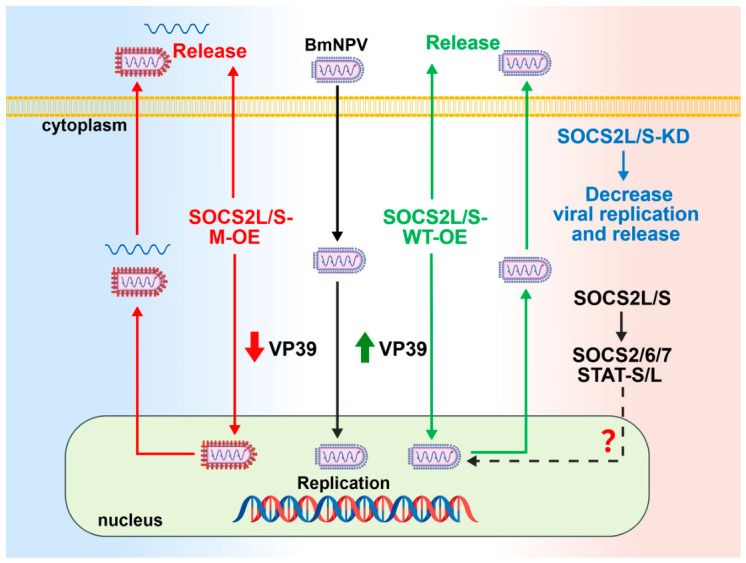
A summary of *SOCS2* regulation of BmNPV proliferation and the SOCS-STAT network. Overexpression of wild-type *SOCS2L/S* (*SOCS2L/S*-WT-OE) promotes BmNPV replication by enhancing viral DNA replication, *VP39* expression (thereby increasing assembly), viral titers, and viral DNA/particle release. Knockdown of *SOCS2L/S* (*SOCS2L/S*-KD) suppresses all these processes. Overexpression of SH2 domain mutants (*SOCS2L/S*-M-OE) neutralizes viral DNA replication to control levels, yet reduces *VP39* expression and titers below control levels, while unexpectedly increasing the release of viral DNA/defective particles. The question mark (?) denotes: *SOCS2L/S* transcriptionally regulate SOCS-STAT network members, which may modulate JAK-STAT signaling and viral replication, a possibility requiring further validation. Green arrows indicate promotion; red arrows indicate inhibition. *SOCS2*, suppressor of cytokine signaling 2. BmNPV, Bombyx mori nucleopolyhedrovirus. This diagram was created using BioGDP.com (https://www.BioGDP.com/#/). (accessed on 17 April 2026).

The present study demonstrates that silkworm *SOCS2* isoforms promote BmNPV proliferation by differentially regulating multiple stages of the viral life cycle with both SH2-dependent and -independent mechanisms (Figure 8). They also transcriptionally modulate the SOCS-STAT network either alone or in cooperation with BmNPV, a novel finding that may significantly impact both the JAK-STAT pathway and viral infection. In addition, *SOCS2* isoforms possess specialized and temporally distinct functions, wherein *SOCS2L* exhibits greater potency than *SOCS2S*.

These findings derive from an in vitro cell model and are limited to steady-state mRNA level analysis. Future work will focus on in vivo validation and protein-level analysis of alterations in the JAK-STAT pathway. A key unresolved question is how *SOCS2* transcriptionally modulates the SOCS-STAT network, possibly through differential regulation of *STAT-L/S*, modulation of their mRNA or protein stability, or indirect effects via unknown transcriptional regulators or chromatin remodelers. All of these possibilities warrant further investigation.

Many insects offer significant economic and nutritional value, such as silkworms [37], mealworms [38,39], honeybees [40,41,42,43,44,45], and various edible insects [46]. Apoptosis is a classic antiviral defense with discoveries continually emerging, such as the interaction of BmNPV p53 with voltage-dependent anion channel 2 (VDAC2) [47], modulation of apoptosis through uric acid metabolism [48,49], reactive oxygen species (ROS) [50], or pattern recognition receptors [51]. In contrast, the antiviral JAK-STAT pathway in insects has only recently begun to receive attention. Our study demonstrates multiple proviral functions of silkworm *SOCS2* and suggests that it may modulate the mRNA expression of the SOCS-STAT network members, implicating it as a potentially important target for viral hijacking to evade host immunity and opening new avenues for bolstering immunity in beneficial insects.

## Data Availability

The original contributions presented in this study are included in the Supplementary Materials. Further inquiries can be directed to the corresponding author.

## Supplementary Materials

The following supporting information can be downloaded at: https://www.mdpi.com/article/10.3390/insects17050503/s1, Table S1: List of primers.
