# Supplementary material for: Silkworm SOCS2 Differentially Promotes Multiple Steps of BmNPV Proliferation and Modulates the mRNA Expression of SOCS-STAT Network Components
Source: Insects. 2026 May 15;17(5):503. doi: 10.3390/insects17050503 (PMC13206853; doi:10.3390/insects17050503)
Supplement: Supplementary file 1 [file insects-17-00503-s001.zip › insects-4304567-supplementary.pdf]

Table S1. List of primers.

Primer name	Primer sequences (5'→3')
SOCS2S-F	CGCGGATCCATGGTGGTTAAACTACCAGATGC（*Bam*H I）
SOCS2S-R	CCGCTCGAGGATCGAGTACGGATATTCGCC（*Xho* I）
SOCS2S-Fm	GTGCAAGACTCTGGAGATAGAA
SOCS2S-Rm	TCCAGAGTCTTGCACGACAAAA
SOCS2L-F	CGCGGATCCATGTGCTTAATTAAAAGGCGAGAG（*Bam*H I）
SOCS2L-R	CCGCTCGAGGATCGAGTACGGATATTCGCCTA（*Xho* I）
SOCS2L-Fm	GTCGTGCAAGACTCTGGAGATA
SOCS2L-Rm	TCCAGAGTCTTGCACGACAAAA
SOCS2S-qF	ATGGTGGTTAAACTACCAGATGCG
SOCS2S-qR	GCGCGAGTGATACTCGCAATTG
SOCS2L-qF	GCTGTACAAGGGATCCATGTGC
SOCS2L-qR	AGAGGCACACCTACTTCCAAGC
α-tubulin-qF	CTCCCTCCTCCATACCCT
α-tubulin-qR	ATCAACTACCAGCCACCC
ds*GFP*-F	*TAATACGACTCACTATAGGG*CGACGTAAACGGCCACAAGT
ds*GFP*-R	*TAATACGACTCACTATAGGG*ATGGGGGTGTTCTGCTGGTAG
ds*SOCS2S*-F	*TAATACGACTCACTATAGGG*GGTTAAACTACCAGATGCGTGC
ds*SOCS2S*-R	*TAATACGACTCACTATAGGG*CATGTACCTAGCCAACGGTCT
ds*SOCS2L*-F	*TAATACGACTCACTATAGGG*GACTCTTGCAGCGCGAATTAC
ds*SOCS2L*-R	*TAATACGACTCACTATAGGG*GCCAACGGTCTGTCACAATCT

Note: The underlined bases indicate sites for restriction endonucleases. The italic bases indicate the T7 promoter.
